# In Silico Assessment of Chemical Disinfectants on Surface Proteins Unveiled Dissimilarity in Antiviral Efficacy and Suitability towards Pathogenic Viruses

**DOI:** 10.3390/ijms25116009

**Published:** 2024-05-30

**Authors:** Diaiti Zure, Meng-Hau Sung, Abdul Rahim, Hsion-Wen Kuo

**Affiliations:** Department of Environmental Science and Engineering, Tunghai University, Taichung 407224, Taiwan; zdiaiti84@gmail.com (D.Z.); mhsung@thu.edu.tw (M.-H.S.); abd.rahimling89@gmail.com (A.R.)

**Keywords:** antiviral efficacy, rutin, eco-pharmaco-economic, in silico assessment, sustainability

## Abstract

Viral pathogens pose a substantial threat to public health and necessitate the development of effective remediation and antiviral strategies. This short communication aimed to investigate the antiviral efficacy of disinfectants on the surface proteins of human pathogenic viruses. Using in silico modeling, the ligand-binding energies (LBEs) of selected disinfectants were predicted and combined with their environmental impacts and costs through an eco-pharmaco-economic analysis (EPEA). The results revealed that the binding affinities of chemical disinfectants to viral proteins varied significantly (*p* < 0.005). Rutin demonstrated promising broad-spectrum antiviral efficacy with an LBE of −8.49 ± 0.92 kcal/mol across all tested proteins. Additionally, rutin showed a superior eco-pharmaco-economic profile compared to the other chemicals, effectively balancing high antiviral effectiveness, moderate environmental impact, and affordability. These findings highlight rutin as a key phytochemical for use in remediating viral contaminants.

## 1. Introduction

The persistent emergence and re-emergence of viral pathogens pose a significant challenge to global public health, emphasizing the need for effective and sustainable antiviral strategies [1,2,3]. The development of antiviral resistance, as observed in HIV and seasonal influenza strains, underscores the adaptability of viruses and the limitations of the existing therapeutic approaches [4,5,6]. The recent COVID-19 pandemic has highlighted the urgent need for robust, sustainable, and accessible antiviral therapies [7,8]. Although chemical disinfectants, antiviral drugs, and phytochemicals have shown promise in targeting various stages of viral infections [9,10], the development of resistance and environmental challenges pose significant hurdles to their sustainability [11,12]. This short communication aimed to assess the antiviral efficacy and sustainability of chemical disinfectants against major human pathogenic viruses using the computational evaluations of disinfectant categories targeting viral structural proteins. We introduced an eco-pharmaco-economic analysis, a novel approach that considers the trifecta of efficacy, environmental safety, and cost to guide the selection and/or development of effective and sustainable viral control strategies. This approach addresses the immediate need for potent antiviral agents while considering the broader implications for public health policy and research, setting the stage for a more resilient global health infrastructure in the face of pandemics.

## 2. Results and Discussion

**Figure 1:**
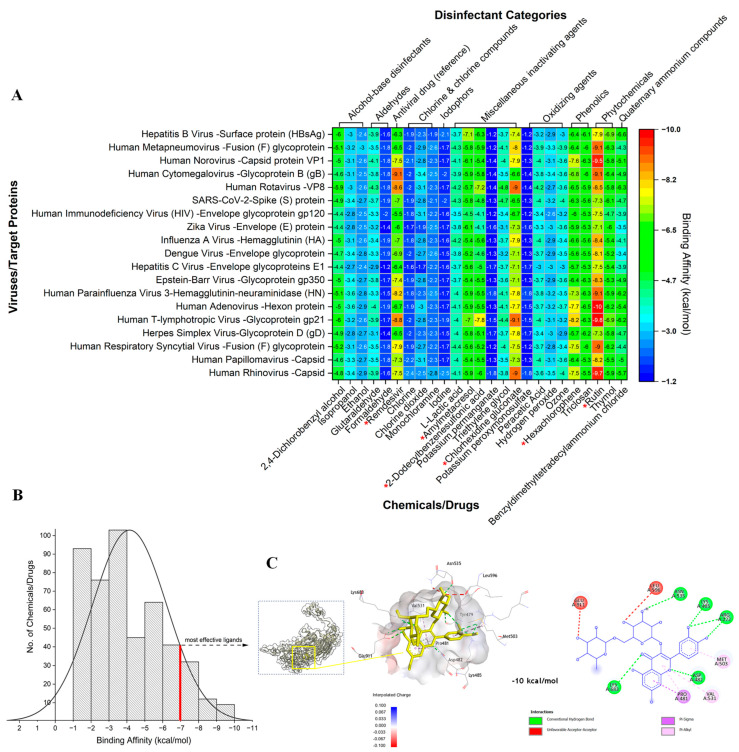
Heat map showing the ligand-binding energy (LBE) of the studied disinfectants with their target external protein (**A**), the distribution of disinfectant LBEs compared with the best LBE (−7 kcal/mol) (**B**), and the molecular interactions between rutin and the hexon protein of human adenovirus (**C**). Red stars (*) denote the most effective disinfectants predicted in this study.

The molecular docking analyses revealed significant variations (*p* < 0.05) in ligand-binding energies (LBEs) between various chemicals (i.e., disinfectants/antiviral drugs) and viral external proteins (Figure 1A). The majority (66.67%) of the tested chemicals/drugs displayed fair binding affinities (LBEs above the threshold, i.e., >−7 kcal/mol, Figure 1B), suggesting the potential further denaturation or disruption of viral external proteins.

Among the tests, rutin (−8.49 ± 0.92 kcal/mol), remdesivir (−7.85 ± 0.73 kcal/mol), hexachlorophene (−7.63 ± 0.31 kcal/mol), chlorhexidine gluconate (−7.81 ± 0.68 kcal/mol), amylmetacresol (−7.05 ± 0.07 kcal/mol), and 2-dodecylbenzenesulfonic acid (−7.50 ± 0.42 kcal/mol) showed promising affinities (Figure 1A). Notably, rutin exhibited broad-spectrum activities against all the tested proteins, with binding affinities ranging from −7.1 to −10 kcal/mol. Further theoretical molecular interactions between rutin and the hexon protein of human adenovirus revealed a complex network of hydrogen bonds and hydrophobic interactions (Figure 1C), indicating a robust inhibitory mechanism with broad applicability. Moreover, the binding affinities of the tested chemicals were somewhat low and narrowly distributed for certain disinfectant categories (i.e., chlorine and chlorine compounds), resulting in higher efficacy for some categories (Figure 1A,B). Consequently, phytochemicals exhibited higher efficiency than antiviral drugs, surpassing that of phenolics, alcohol-based disinfectants, quaternary ammonium compounds, and other inactivating agents (Figure 1A). This observation highlights the potential of phytochemicals to disrupt the viral external surface proteins. Additionally, some viruses (i.e., human cytomegalovirus, human T-lymphotropic virus, human rotavirus, and human parainfluenza virus) show high susceptibility to chemicals within the aforementioned categories, suggesting vulnerabilities in their surface proteins. These results indicate that traditional disinfectants (i.e., chlorine and chlorine compounds) may be less effective than phytochemicals in remediating and treating human pathogenic viruses. 

**Figure 2:**
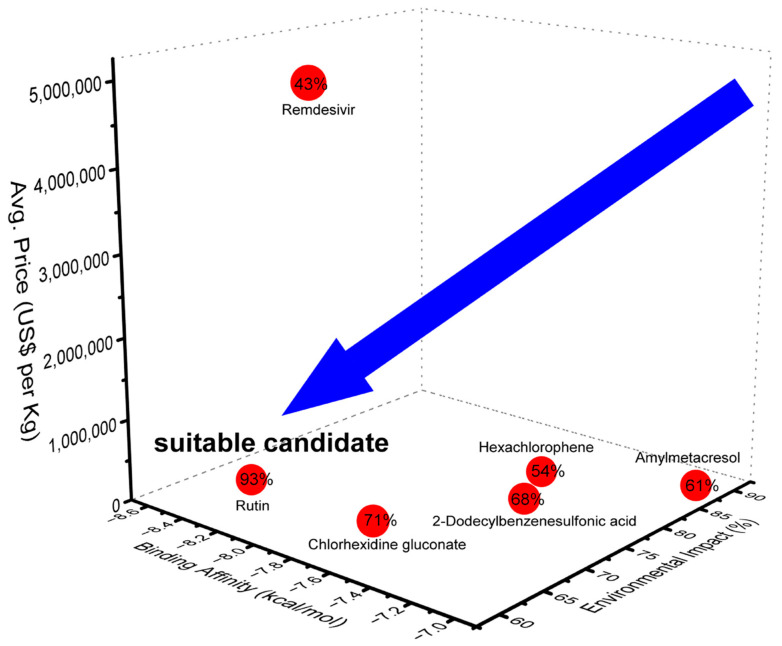
Proposed eco-pharmaco-economic analysis (EPEA) of the most effective disinfectants. The EPEA presents the pricing affordability (i.e., lower cost indicates better affordability), environmental impacts (i.e., safe: ≤33.33%, mild: 33.34–66.66%, and danger: ≥66.67%), and antiviral efficacy (i.e., binding affinity > −7 kcal/mol is better) of the chemical disinfectants. The EPEA ranks the chemicals in order of their overall performance (O), where a higher percentage indicates a suitable candidate (i.e., antiviral effective and sustainable chemical). Red circles denote EPEA scores, while blue arrow points to suitable candidates.

Furthermore, the eco-pharmaco-economic analysis (EPEA) of the chemicals with promising LBEs (Figure 2) revealed that rutin was the most favorable candidate (93%), demonstrating a balance between high antiviral efficacy (−8.49 kcal/mol), moderate environmental impact (66%), and reasonable cost (USD 33.52/kg). This harmonious balance makes rutin a promising candidate for widespread antiviral applications. Conversely, despite its known antiviral properties, remdesivir faces limitations due to its comparatively moderate efficacy (−7.85 kcal/mol) and higher cost (USD 5.2 M/kg). The EPEA ranked the remaining chemicals from most to least favorable based on a composite score of their LBEs, environmental impacts, and costs in the following order: chlorhexidine gluconate, 2-dodecylbenzenesulfonic acid, amylmetacresol, and hexachlorophene (Figure 2). This ranking underscores the need for a judicious approach to both the development and deployment of antiviral agents, highlighting the importance of integrating LBEs with environmental and economic considerations to select suitable chemicals for crafting sustainable antiviral strategies and remediating viral contaminants.

It has been reported that effective disinfectants are critical in curbing viral transmission to susceptible populations [13]. While previous research has extensively characterized the biocidal properties of disinfectants, understanding their molecular interactions with viral components remains a key area of investigation [13,14]. The ligand-binding energy (LBE) between a disinfectant and viral protein is a critical determinant of antiviral efficacy, indicating the potential for stable and strong complex formation [15]. Recent findings have shed light on the varying antiviral efficacy of disinfectants. Although some chemicals demonstrate high effectiveness by strongly denaturing or disrupting the target surface proteins, others exhibit moderate effectiveness, which may be attributed to their lower binding affinities or less precise targeting of the complex and variable structures of viral proteins [16,17]. The chemical disinfectants studied could potentially exhibit diverse modes of action against viral surface proteins, including direct (i.e., protein denaturation and membrane disruption by phytochemicals, aldehydes, and alcohol-base disinfectants), indirect (i.e., oxidative damage by hydrogen peroxide and chlorine), and multi-faceted (i.e., both oxidative damage and direct binding interactions by peracetic acid and ozone). Binding affinity can provide valuable insights into the antiviral mechanisms of disinfectants. Developing approaches for evaluating the indirect mode of action for some disinfectant categories (i.e., oxidative damage by agents such as hydrogen peroxide, chlorine, and potassium permanganate) is a prerequisite for fully comprehending their antiviral efficacy. The promising efficacies of some chemicals (i.e., rutin, remdesivir, hexachlorophene, chlorhexidine gluconate, amylmetacresol, and 2-dodecylbenzenesulfonic acid) against specific surface proteins (i.e., glycoprotein gB, glycoprotein gp21, VP8, and hemagglutinin HA) are promising avenues for targeted remediation and therapeutic intervention. These proteins are paramount for viral attachment, entry, replication, and assembly, making them prime targets for a wide range of chemical disinfectants. Moreover, the exceptional broad-spectrum efficacy of rutin across all the tested surface proteins may be attributed to its favorable binding energies and specific molecular interactions with the amino acid residues of viral proteins. These promising characteristics are supported by our recent study [10], which elucidated the molecular basis of rutin antiviral activities, demonstrating strong affinities to the essential proteins of both MS2 and T4 viruses. Phytochemicals such as rutin serve as naturally occurring disinfectants. Their diverse chemical structures may facilitate multiple interactions with viral targets, potentially explaining their heightened efficacy compared to conventional disinfectants, such as phenolics or alcohol-based agents, which typically exhibit broader, less targeted mechanisms of action [18,19,20]. In contrast to phytochemicals, most conventional disinfectants with lower binding affinities rely on nonspecific mechanisms such as protein denaturation or membrane disruption, which may not provide the same level of targeted antiviral activity [21]. This distinction highlights the importance of strategic disinfectant selection, which favors chemicals with higher specificity and binding affinity. Such an approach is not only promising for the development of antiviral drugs but also has potential applications in environmental remediation, where the targeted binding of compounds to specific proteins could enhance the degradation or removal of pollutants.

Moreover, the contemporary landscape of antiviral agent development and deployment necessitates evaluations encompassing both efficacy and sustainability [22]. Our proposed eco-pharmaco-economics approach integrates antiviral efficacy with environmental sustainability and cost-effectiveness. This provides a holistic framework for assessing the viability of antiviral chemicals. This approach has become particularly relevant given the mounting environmental concerns and economic constraints confronting healthcare systems globally. Prior studies have focused on the pharmacodynamics and pharmacokinetics of antiviral agents, often overlooking the extensive ecological and economic implications linked to their widespread application [8,23,24]. Through the eco-pharmaco-economic analysis, nuanced distinctions among the leading antiviral chemicals were revealed. This complexity underscores the challenge of selecting effective and sustainable antiviral strategies. Rutin is an exemplary antiviral agent in eco-pharmaco-economics because of its exceptional balance between high antiviral efficacy, moderate environmental impact, and affordability. The pronounced efficacy of rutin underscores its suitability as a prime candidate for sustainable antiviral solutions. This context is significant when considering the environmental challenges of agro-industrial biowastes (e.g., air emissions, effluents, and solid waste) [25]. These biowastes are rich in phenolic compounds (i.e., phenolic acids and flavonoids), highlighting both a sustainability challenge and an opportunity for innovation. By extracting these compounds using non-toxic solvents [25], pollution sources are transformed into valuable assets. This process reduces environmental harm, improves human health, and increases economic value. In contrast, the low EPEA profile of remdesivir emphasizes the necessity for a balanced approach that considers not only the biological effectiveness of antiviral agents but also their ecological and economic implications. The EPEA offers a promising framework for evaluating antiviral chemicals, advocating sustainable interventions, and emphasizing an integrated evaluation framework. Embracing these principles enhances our capacity to combat viral pathogens, ensures sustainable strategies, and contributes to a more resilient global health landscape through informed public health policies and antiviral research decisions.

## 3. Materials and Methods

A multi-faceted computational approach that integrated in silico modeling with eco-pharmaco-economic analysis (EPEA) to evaluate the antiviral efficacy and sustainability of various chemical disinfectants against external surface proteins was used. The disinfectants were chosen based on their widespread use, established antiviral properties, and relevance to the current public health challenges (Table S1). The viruses prevalent in Taiwan were selected to ensure the regional applicability of our findings and address specific public health concerns (Table S2). By inhibiting surface proteins essential for the viral life cycle, viral attachment and entry can be reduced. Although some surface proteins are not typically targeted for replication inhibition, disrupting them can significantly impact their functional roles and overall infectivity.

Molecular docking simulations were performed using PyRx v.0.8 (Scripps Research Institute, La Jolla, CA, USA), QuickVina2 [26], and BIOVIA Discovery Studio 21.1 (Dassault Systèmes, Waltham, MA, USA), following a modified approach based on Kuo et al. [27] (Text S1). To simulate practical disinfection scenarios, we employed excess ligand conditions, reflecting the higher concentrations of chemical disinfectants typically applied relative to the viral surface proteins. Traditional 1:1 protein–ligand docking does not adequately model these conditions; therefore, we implemented blind docking to identify up to nine potential binding positions for each ligand at various protein-binding sites [26]. This approach allowed us to simulate multiple ligands binding with a protein, better reflecting the high-concentration conditions of disinfection processes. Optimal binding positions were determined based on the lowest binding energy (LBE), with additional positions representing potential interactions under high ligand conditions. The antiviral efficiency of various disinfectants could be comprehensively assessed by complementing binding affinity studies with the analyses of direct, indirect, and multi-faceted actions. Given that predicting the indirect mode of action is lacking, previous studies have primarily focused on binding interactions, providing a foundational approach to understanding how disinfectants function, even for those that act as denaturation reagents [28,29]. Binding affinity studies are instrumental in evaluating the interactions between disinfectants and viral proteins, providing crucial insights into their antiviral mechanisms. Incorporating molecular dynamics (MD) simulations and experimental validations to enhance our understanding of the effects of ligand concentration on binding affinity and molecular interactions could be implemented in future work. 

We explored the functional roles of the target proteins using the QuickGO web-based tool (European Bioinformatics Institute (EMBL-EBI), Hinxton, Cambridgeshire, UK). The statistical analyses of variations in LBEs were conducted using the GraphPad Prism v.9.5 (GraphPad Software, Inc., San Diego, CA, USA). Our EPEA framework integrates antiviral efficacy with environmental impact and cost-effectiveness, employing the Weighted Score Method (WSM) and Multicriteria Decision Analysis (MCDA) to evaluate disinfectants across these dimensions (Text S2). The environmental safety profiles were assessed using the US EPA Estimation Program Interface Suite v.4.11 (United States Environmental Protection Agency (US EPA), Washington, DC, USA) [30], which provides a comprehensive evaluation of the potential ecological impact. Cost data for the disinfectants were obtained from extensive market research and public pricing information (Table S3), ensuring that the economic analysis was grounded in real-world data.

## 4. Conclusions

This study presents an innovative framework that demonstrates the antiviral efficacy and sustainability of chemical disinfectants against major pathogenic viruses in humans. Noteworthy disclosures have highlighted the promising broad-spectrum efficacy of rutin and the elevated potential of phytochemicals for disrupting the surface proteins of pivotal human pathogenic viruses. The newly proposed eco-pharmaco-economic analysis approach seems to better evaluate the applicability of disinfectants by considering multiple key aspects (i.e., efficacy, costs, and safety) for minimizing possible biases (e.g., high efficacy but high costs and/or safety concerns). The collective significance of these findings lies in their potential for selecting and/or developing sustainable antiviral agents with enhanced effectiveness and broad-spectrum capabilities for remediating viral contaminants. By integrating theoretical chemical efficacy, environmental considerations, and economic feasibility, this communication lays the foundation for more potent antiviral interventions that harmonize efficacy with sustainability. These outcomes have profound implications for future research, emphasizing the importance of strategic disinfection choices and targeted antiviral advancements.

## Data Availability

All supporting data can be found in the Supplementary Files.

## Supplementary Materials

The following supporting information can be downloaded at: https://www.mdpi.com/article/10.3390/ijms25116009/s1.

## References

[1] Guo J., Ma X., Xu X., Guo Y., Li B., Wang M., Wang Y. (2022). Zika Virus Infection and Development of Drug Therapeutics. Appl. Microbiol..

[2] Ouyang Y., Chen Y., Shang J., Sun S., Wang X., Huan S., Xiong B., Zhang X.-B. (2023). Virus-like Plasmonic Nanoprobes for Quick Analysis of Antiviral Efficacy and Mutation-Induced Drug Resistance. Anal. Chem..

[3] Sangkham S. (2021). A Review on Detection of SARS-CoV-2 RNA in Wastewater in Light of the Current Knowledge of Treatment Process for Removal of Viral Fragments. J. Environ. Manag..

[4] Adwani C. (2021). Antimalarial and Antiviral Drugs: A Review of Trials and Effectiveness in Treating COVID-19. Biosci. Biotechnol. Res. Commun..

[5] Clarridge K.E., Blazkova J., Einkauf K., Petrone M., Refsland E.W., Justement J.S., Shi V., Huiting E.D., Seamon C.A., Lee G.Q. (2018). Effect of Analytical Treatment Interruption and Reinitiation of Antiretroviral Therapy on HIV Reservoirs and Immunologic Parameters in Infected Individuals. PLoS Pathog..

[6] Fischer W.A., Uyeki T.M., Tauxe R.V. (2015). Ebola Virus Disease: What Clinicians in the United States Need to Know. Am. J. Infect. Control.

[7] Comunale B.A., Larson R.J., Jackson-Ward E., Singh A., Koback F.L., Engineer L.D. (2023). The Functional Implications of Broad Spectrum Bioactive Compounds Targeting RNA-Dependent RNA Polymerase (RdRp) in the Context of the COVID-19 Pandemic. Viruses.

[8] Wang R., Luo J., Li C., Chen J., Zhu N. (2023). Antiviral Drugs in Wastewater Are on the Rise as Emerging Contaminants: A Comprehensive Review of Spatiotemporal Characteristics, Removal Technologies and Environmental Risks. J. Hazard. Mater..

[9] Ashokkumar S., Kaushik N.K., Han I., Uhm H.S., Park J.S., Cho G.S., Oh Y.-J., Shin Y.O., Choi E.H. (2023). Persistence of Coronavirus on Surface Materials and Its Control Measures Using Nonthermal Plasma and Other Agents. Int. J. Mol. Sci..

[10] Zure D., David Kuo H.-W., Drizo A. (2024). Insights of Phytoremediation Mechanisms for Viruses Based on In-Vitro, In-Vivo and In-Silico Assessments of Selected Herbal Plants. Chemosphere.

[11] Bivacqua R., Barreca M., Spanò V., Raimondi M.V., Romeo I., Alcaro S., Andrei G., Barraja P., Montalbano A. (2023). Insight into Non-Nucleoside Triazole-Based Systems as Viral Polymerases Inhibitors. Eur. J. Med. Chem..

[12] Pattnaik G.P., Chakraborty H. (2020). Entry Inhibitors: Efficient Means to Block Viral Infection. J. Membr. Biol..

[13] Castaño N., Cordts S.C., Kurosu Jalil M., Zhang K.S., Koppaka S., Bick A.D., Paul R., Tang S.K.Y. (2021). Fomite Transmission, Physicochemical Origin of Virus–Surface Interactions, and Disinfection Strategies for Enveloped Viruses with Applications to SARS-CoV-2. ACS Omega.

[14] Marquès M., Domingo J.L. (2021). Contamination of Inert Surfaces by SARS-CoV-2: Persistence, Stability and Infectivity. A Review. Environ. Res..

[15] Ren S., Fraser K., Kuo L., Chauhan N., Adrian A.T., Zhang F., Linhardt R.J., Kwon P.S., Wang X. (2022). Designer DNA Nanostructures for Viral Inhibition. Nat. Protoc..

[16] Borisevich S.S., Zarubaev V.V., Shcherbakov D.N., Yarovaya O.I., Salakhutdinov N.F. (2023). Molecular Modeling of Viral Type I Fusion Proteins: Inhibitors of Influenza Virus Hemagglutinin and the Spike Protein of Coronavirus. Viruses.

[17] Dassanayake M.K., Khoo T.-J., Chong C.H., Di Martino P. (2022). Molecular Docking and In-Silico Analysis of Natural Biomolecules against Dengue, Ebola, Zika, SARS-CoV-2 Variants of Concern and Monkeypox Virus. Int. J. Mol. Sci..

[18] Ghildiyal R., Prakash V., Chaudhary V.K., Gupta V., Gabrani R. (2020). Phytochemicals as Antiviral Agents: Recent Updates. Plant-Derived Bioactives.

[19] Kim E.-H., Lee B.W., Ryu B., Cho H.M., Kim S.-M., Jang S.-G., Casel M.A.B., Rollon R., Yoo J.-S., Poo H. (2022). Inhibition of a Broad Range of SARS-CoV-2 Variants by Antiviral Phytochemicals in HACE2 Mice. Antivir. Res..

[20] Umar A.K., Zothantluanga J.H., Aswin K., Maulana S., Sulaiman Zubair M., Lalhlenmawia H., Rudrapal M., Chetia D. (2022). Antiviral Phytocompounds “Ellagic Acid” and “(+)-Sesamin” of *Bridelia retusa* Identified as Potential Inhibitors of SARS-CoV-2 3CL pro Using Extensive Molecular Docking, Molecular Dynamics Simulation Studies, Binding Free Energy Calculations, and Bioactivity Prediction. Struct. Chem..

[21] Farooq S., Ngaini Z. (2021). Natural and Synthetic Drugs as Potential Treatment for Coronavirus Disease 2019 (COVID-2019). Chem. Afr..

[22] Spengler J.R., Welch S.R., Deval J., Gentry B.G., Brancale A., Carter K., Moffat J., Meier C., Seley-Radtke K.L., Schang L.M. (2023). Meeting Report: 35th International Conference on Antiviral Research in Seattle, Washington, USA–March 21–25, 2022. Antivir. Res..

[23] Eryildiz B., Ozgun H., Ersahin M.E., Koyuncu I. (2022). Antiviral Drugs against Influenza: Treatment Methods, Environmental Risk Assessment and Analytical Determination. J. Environ. Manag..

[24] Zhang J., Zhu M., Wang Q., Yang H. (2023). The Combined Use of Copper Sulfate and Trichlorfon Exerts Stronger Toxicity on the Liver of Zebrafish. Int. J. Mol. Sci..

[25] Zalazar-García D., Torres E., Rodriguez-Ortiz L., Deng Y., Soria J., Bucalá V., Rodriguez R., Mazza G. (2020). Cleaner and Sustainable Processes for Extracting Phenolic Compounds from Bio-Waste. J. Environ. Manag..

[26] Alhossary A., Handoko S.D., Mu Y., Kwoh C.K. (2015). Fast, Accurate, and Reliable Molecular Docking with QuickVina 2. Bioinformatics.

[27] Kuo H.-W.D., Zure D., Lin C.-R. (2023). Occurrences of Similar Viral Diversity in Campus Wastewater and Reclaimed Water of a University Dormitory. Chemosphere.

[28] Lin N., Verma D., Saini N., Arbi R., Munir M., Jovic M., Turak A. (2021). Antiviral Nanoparticles for Sanitizing Surfaces: A Roadmap to Self-Sterilizing against COVID-19. Nano Today.

[29] Yamamoto Y., Nakano Y., Murae M., Shimizu Y., Sakai S., Ogawa M., Mizukami T., Inoue T., Onodera T., Takahashi Y. (2023). Direct Inhibition of SARS-CoV-2 Spike Protein by Peracetic Acid. Int. J. Mol. Sci..

[30] US EPA (2024). Estimation Programs Interface SuiteTM for Microsoft® Windows, v 4.11.

