# Supplementary material for: In Silico Assessment of Chemical Disinfectants on Surface Proteins Unveiled Dissimilarity in Antiviral Efficacy and Suitability towards Pathogenic Viruses
Source: Int J Mol Sci. 2024 May 30;25(11):6009. doi: 10.3390/ijms25116009 (PMC11172749; doi:10.3390/ijms25116009)
Supplement: Supplementary file 1 [file ijms-25-06009-s001.zip › ijms-3014090-supplementary.pdf]

Supplementary

Text S1 Modified molecular docking and function predictions of viral proteins

Computational docking tools, including PyRx (v.08), QuickVina2, and BIOVIA Discovery Studio (v.21.1), were used for molecular docking and interaction analyses following the modified approach of Kuo et al. (2023)[1]. The modified approach involved the blind docking of each target structural protein with the antiviral drug remdesivir using PyRx, and the generated binding configuration was further utilized for batch docking with QuickVina2. The ligand structures of the chemical disinfectants (SDF format) were obtained from the NCBI PubChem database, and the protein structures (PDB format) were obtained from the RCSB PDB database. Remdesivir was used as a benchmark to establish the antiviral efficacy of disinfectants against diverse viruses.

Text S2 Proposed eco-pharmaco-economic analysis (EPEA) of disinfectants

The eco-pharmaco-economic analysis (EPEA) involved a combination of the Weighted Score Method (WSM) and Multicriteria Decision Analysis (MCDA): Six critical environmental and safety profiles were conducted for the most effective chemical disinfectants, employing the US EPA Estimation Program Interface (EPI) Suite (v4.11). These profiles included the atmospheric hydroxylation rate (logOH), soil adsorption coefficient (logKoc), fish biotransformation half-life (HLN), bioconcentration factor (logBCF), bioaccumulation factor (logBAF), and biodegradation

[1] Kuo, H. W. D., Zure, D., & Lin, C. R. (2023). Occurrences of similar viral diversity in campus wastewater and reclaimed water of a university dormitory. Chemosphere, 330, 138713. https://doi.org/10.1016/j.chemosphere.2023.138713

half-life (DT50). The outcomes of these profiles were classified into three tiers (i.e., safe, mild, and danger) according to criteria derived from the established environmental and toxicological benchmarks integrated within the EPI Suite. This stratification facilitated a detailed assessment of their specific ecological and health-related effects, thereby providing a nuanced understanding of the potential risks associated with each profile. For each profile, a categorical scoring system was proposed (safe, 1; mild, 2; and danger, 3). The cumulative scores of these categories were then compared with the maximum possible score (max. categorical score (3) × number of impact profiles (6)). This comparison is expressed as a percentage to ascertain the overall environmental impact, as illustrated in Equation (1):

$$\textit{Environmental Impact (EI, \%)} = (\textit{Tot. Profile Score} / \textit{Max. Assigned Score}) \times 100\% \qquad \text{(Eq.1)}$$

where *EI* utilized the WSM and delineated it into three classifications: safe (≤ 33.33%), mild (33.34-66.66%), and danger (≥66.67%). *Tot. Profile Score* refers to the aggregated scores from the six environmental profiles, while *Max. Assigned Score* indicates the highest possible score.

Moreover, the cost-effectiveness of the prominent disinfectants was evaluated. Prices per kilogram (USD/kg) for the most effective chemicals were sourced from Chemicalbook[2], and the price for remdesivir was acquired from Gilead Sciences[3]. The cumulative performance of each chemical, including affordability (*C*), environmental safety (*S*), and antiviral efficacy (*E*), was ranked in ascending order by using MCDA. This ranking was established to highlight the overall performance (*O*) of each chemical, using proposed Equation (2):

$$O(\%) = wE \times Enorm + wS \times Snorm + wC \times Cnorm \qquad \text{(Eq.2)}$$

[2] Chemicalbook (2024). Chemicalbook. Chemicalbook. url: https://www.chemicalbook.com/

[3] Gilead Sciences (2024). Gilead Sciences. Gilead Sciences Inc. url: https://www.gilead.com/

where *wE*, *wS*, and *wC* signify the weighted contributions of antiviral efficacy, environmental safety, and cost, respectively. Equal weights were allocated to *C*, *S*, and *E* (i.e., $wE + wS + wC = 1$ or $1/3E + 1/3S + 1/3C = 1$) and normalized to a uniform scale (0 to 100) using proposed Equations (3), (4), and (5).

$$Enorm = E - min(E) / max(E) - min(E) \; x \; 100 \quad \text{(Eq.3)}$$

where *E* represents the LBE of a chemical with *min(E)* and *max(E)* denoting the most negative and least negative LBE values in the dataset, respectively. This normalization ensures that a score of 100 corresponds to the highest efficacy (most negative LBE), whereas a score of 0 indicates the lowest efficacy.

$$Snorm = 100 - (S - min(S) / max(S) - min(S) \; x \; 100) \quad \text{(Eq.4)}$$

where *S* refers to the environmental safety of a chemical as determined by its impact assessment percentage. *min(S)* and *max(S)* are the lowest and highest impact percentages in the dataset, indicating the best and worst safety profiles, respectively. A lower impact percentage is synonymous with a better safety profile.

$$Cnorm = 100 - (S - min(C) / max(C) - min(C) \; x \; 100) \quad \text{(Eq.5)}$$

where *C* represents the cost of the chemical. *min(C)* and *max(C)* represent the lowest and highest costs in the dataset, respectively, with lower costs indicating higher affordability. This normalization ensures that a lower cost translates to a higher score, reflecting better affordability.

Table S1 Different categories of disinfectants and their chemicals

Types	Name	PubChemCID
Alcohol-based disinfectants	Ethanol	702
	Isopropanol	3776
	2,4-Dichlorobenzyl Alcohol	15684
Aldehydes	Formaldehyde	712
	Glutaraldehyde	3485
Antiviral drugs (reference)	Remdesivir	121304016
Chlorine and chlorine compounds	Chlorine	24526
	Chlorine Dioxide	24870
	Monochloramine	25423
Iodophors	Iodine	807
Miscellaneous inactivating agents	L-Lactic Acid	107689
	Amylmetacresol	14759
	2-Dodecylbenzenesulfonic Acid	25457
	Potassium Permanganate	516875
	Triethylene Glycol	8172
	Chlorhexidine Gluconate	9552081
Oxidizing agents	Hydrogen Peroxide	784
	Peracetic Acid	6585
	Ozone	24823
	Potassium Peroxymonosulfate	156589072
Phenolic compounds	Triclosan	5564
	Hexachlorophene	3598
Phytochemicals	Rutin	5280805
	Thymol	6989
Quaternary ammonium compounds	Benzyldimethyltetradecylammonium Chloride	8756

Table S2 Surface proteins of pivotal human pathogenic viruses

Virus	Surface proteins	PDB ID
Human Parainfluenza Virus 3	Hemagglutinin-neuraminidase (HN)	1V3B
SARS-CoV-2	Spike (S) protein	6VXX
Human Immunodeficiency Virus (HIV)	Envelope glycoprotein gp120	6MEO
Hepatitis C Virus	Envelope glycoproteins E1	4N0Y
Influenza A Virus	Hemagglutinin (HA)	4WE9
Hepatitis B Virus	Surface protein (HBsAg)	7TUK
Human Papillomavirus	L1 capsid protein	1DZL
Epstein-Barr Virus	Glycoprotein gp350	2H6O
Human Cytomegalovirus	Glycoprotein B (gB)	7KDP
Human T-lymphotropic Virus	Glycoprotein gp21	1MG1
Herpes Simplex Virus	Glycoprotein D (gD)	1JMA
Human Adenovirus	Hexon protein	1P30
Human Rhinovirus	Capsid proteins	1AYM
Human Norovirus	Capsid protein VP1	7MRY
Human Rotavirus	VP8	7C8P
Human Respiratory Syncytial Virus	Fusion (F) glycoprotein	1G2C
Human Metapneumovirus	Fusion (F) glycoprotein	7SEJ
Zika Virus	Envelope (E) protein	5JHM
Dengue Virus	Envelope glycoprotein	4UTC

Table S3 Eco-pharmaco-economic analysis (EPEA) of candidate chemical disinfectants

Disinfectant category	Chemical/drug	Avg. ligand binding energy (kcal/mol)	Environmental impact (%)	Avg. Price (USD/Kg/Vial)	EPEA ranking (%)
Miscellaneous inactivating agents	2-Dodecylbenzenesulfonic acid	-7.5	77.78	100.00	68
	Amylmetacresol	-7.05	88.98	7.00	61
	Chlorhexidine gluconate	-7.81	66.67	17.5	71
Phenolic compounds	Hexachlorophene	-7.63	83.33	12.5	54
Antiviral drug (reference)	Remdesivir	-7.85	61.11	5.2 M*	43
Phytochemicals	Rutin	-8.49	66.67	33.52	93

*M denotes million USD, where 1 vial is equal to 100 mg and priced at USD 520.
